# A FcγRIII-engaging bispecific antibody expands the range of HER2-expressing breast tumors eligible to antibody therapy

**DOI:** 10.18632/oncotarget.2093

**Published:** 2014-06-11

**Authors:** Marc Turini, Patrick Chames, Pierre Bruhns, Daniel Baty, Brigitte Kerfelec

**Affiliations:** ^1^ INSERM, U1068, CRCM, Marseille, France; ^2^ Institut Paoli-Calmettes, Marseille, France; ^3^ Aix-Marseille Université, UM105, Marseille, France; ^4^ CNRS, UMR7258, CRCM, Marseille, France; ^5^ Département d'Immunologie, Laboratoire Anticorps en Thérapie et Pathologie, Institut Pasteur, Paris, France; ^6^ INSERM, U760, Paris, France

**Keywords:** bispecific antibody, breast cancer, FcγRIIIA polymorphism, HER2, trastuzumab

## Abstract

Trastuzumab is established as treatment of HER2^high^ metastatic breast cancers but many limitations impair its efficacy. Here, we report the design of a Fab-like bispecific antibody (HER2bsFab) that displays a moderate affinity for HER2 and a unique, specific and high affinity for FcγRIII. *In vitro* characterization showed that ADCC was the major mechanism of action of HER2bsFab as no significant HER2-driven effect was observed. HER2bsFab mediated ADCC at picomolar concentration against HER2^high^, HER2^low^ as well as trastuzumab-refractive cell lines. *In vivo* HER2bsFab potently inhibited HER2^high^ tumor growth by recruitment of mouse FcγRIII and IV-positive resident effector cells and more importantly, exhibited a net superiority over trastuzumab at inhibiting HER2^low^ tumor growth. Moreover, FcγRIIIA-engagement by HER2bsFab was independent of V/F158 polymorphism and induced a stronger NK cells activation in response to target cell recognition. Thus, taking advantage of its epitope specificity and affinity for HER2 and FcγRIIIA, HER2bsFab exhibits potent anti-tumor activity against HER2^low^ tumors while evading most of trastuzumab Fc-linked limitations thereby potentially enlarging the number of patients eligible for breast cancer immunotherapy.

## INTRODUCTION

Human epidermal growth factor receptor 2 (HER2) is involved in complex signaling pathways controlling cell growth, survival and proliferation depending on the triggered signaling cascades [1]. Highly overexpressed in 20-25% of breast cancers, HER2 is associated with aggressive disease, increased metastasis potential and poor clinical outcome. The humanized monoclonal antibody trastuzumab (Herceptin^®^), first agent used for targeting HER2, is still standard of care as single agent [2] and in combination with chemotherapy in both early-stage and metastatic breast cancers strongly overexpressing HER2 [3, 4]. However, despite its irrefutable benefit, a significant subset of patients (~70%) with metastatic disease shows a resistance *ab initio* to trastuzumab as single agent and the majority of treated patients develop resistance within one year of treatment [5, 6]. Therefore, primary and acquired resistances to trastuzumab treatment represent an important clinical challenge. Moreover, up to now, the guidelines for trastuzumab treatment eligibility exclude patients with tumors displaying an HER2 immunohistochemistry (IHC) score of 1+/2+.

Trastuzumab exerts its anti-tumor activity via the blockade of constitutive HER2 signaling and the recruitment of FcR expressing immune effector cells responsible for antibody-dependent-cell cytotoxicity (ADCC) [7]. Although the exact contribution of each of these mechanisms *in vivo* is difficult to assess, pre-clinical studies provide evidence of the importance of ADCC in trastuzumab-based therapy [8-10]. The increased number of tumor-infiltrated NK cells observed in tumor tissue after trastuzumab treatment also supports the hypothesis of immune cells recruitment by the antibody [11, 12]. Importantly, FcγRIIIA-158 polymorphism has been shown to significantly influence the efficacy of trastuzumab in breast cancer patients [13]. Finally, Park *et al* [14] recently suggested a contribution of an adaptive immune response involving CD8^+^ T cells, dependent on the initial antibody-triggered innate response through the production of cytokines and/or danger signals by FcR^+^ cells. However, besides FcγRIIIA-158 polymorphism, competition with endogenous IgGs and engagement of inhibitory antibody receptors (FcγRIIB) have been demonstrated to drastically hinder its capacity to mediate efficient ADCC.

Consequently, tremendous efforts are ongoing either to improve the clinical efficacy of trastuzumab or to develop new strategies [15-20]. A promising alternative is the design of bispecific antibodies (bsAb) able to efficiently recruit and activate effector cells at the tumor site. After a first craze in the 90s stopped by inconsistent clinical response and immunotoxicity, a revival of interest for bispecific antibodies has emerged from the evolution in antibody engineering. This led to the development of a large number and a wide variety of bispecific formats based on either IgG or non-IgG scaffolds [21, 22]. Although retargeting of various cytotoxic effector cells is exploited, many bispecific antibodies aim at activating T-cells based on their numeric superiority and their high intrinsic toxicity, some of them being currently under clinical investigations [23-25].

FcγRIIIA positive cells are however interesting to target. In addition to their intrinsic capability to attack tumors, NK cells are not affected by the various mechanisms put in place by tumor cells to escape their recognition by T cells. FcγRIIIA is also expressed on monocytes and macrophages [26] that are important actors of anti tumor immunity [27]. Moreover, in contrast to CD3 targeting, FcγRIIIA targeting does not induce the recruitment and activation of Treg cells, a subset of cells able to downregulate the antitumor immunity. However, despite very encouraging *in vitro* or pre-clinical results, limited clinical data are available on the efficacy of FcγRIII-targeting bispecific antibodies [28] and thus far, only one antibody, a bispecific TandAb targeting CD30 and FcγRIIIA [29] is ongoing a clinical study [NCT01221571].

In a previous study [30], we designed a bispecific antibody based on the natural affinity of human CH1 and Cκ IgG domains as a heterodimerization motif and the unique structural and functional properties of llama single domain antibodies.

In this study, we have exploited the modular structure of the bsFab format to produce a Fab-like bispecific antibody (HER2bsFab) targeting binding sites on HER2 and FcγRIIIA different from those targeted by trastuzumab and conventional IgGs. A side by side comparison of HER2bsFab with trastuzumab has been conducted *in vitro* and in a mouse model to characterize its anti-tumor efficacy against high- and low-HER2-overexpressing, as well as trastuzumab-refractive breast cancer tumors.

## RESULTS

### HER2bsFab binds simultaneously to HER2 and FcγRIIIA

**Figure 1:**
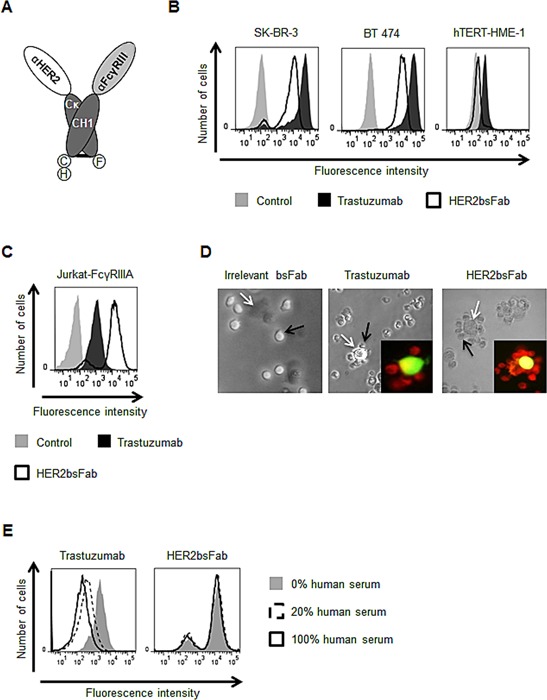
HER2bsFab simultaneously binds to cell surface HER2 and FcγRIIIA A) Schematic representation of the assembled HER2bsFab. Dark grey, Cκ and CH1 constant domains of human IgG1; Light grey, anti-FcγRIII sdAb (clone CD16.21); White, anti-HER2 sdAb (clone HER2.C7b); C, 6H and F, c-myc, hexahistidine and Flag Tag respectively. Binding of biotinylated HER2bsFab and trastuzumab (100 nM) to B) HER2-positive (SK-BR-3, BT 474), HER2-negative (hTERT-HME-1) or C) FcγRIIIA expressing Jurkat-huFcγRIIIA cells was analyzed by flow cytometry. PE-labeled streptavidin was used for detection. D) Rosette forming cells assay: CFSE labeled SK-OV-3 (white arrows) and Red CMTPX labeled Jurkat-huFcγRIIIA (black arrows) cells were co-cultured with irrelevant bsFab, HER2bsFab or trastuzumab (40 nM) and rosette formation was observed by optical and fluorescent microscopy. Representative images of different conditions are shown. E) Competition assay with endogenous IgG: Jurkat-huFcγRIIIa cells were incubated with biotinylated HER2bsFab (50 nM) or trastuzumab (200 nM) in presence of various concentrations of human serum. Bound antibodies were detected using PE-labeled streptavidin.

Based on the modular nature of the previously described compact and linker-free format [30], we designed a bsFab (HER2bsFab) targeting HER2-expressing cancer cells and FcγRIII positive effector cells (Fig. 1A). HER2bsFab was expressed in the periplasm of *E. coli* and purified to homogeneity by a two-step affinity chromatography procedure. HER2bsFab was produced at high yield (2-4 mg/L) and demonstrated a high stability as no significant decrease of binding was observed on SK-OV-3 or Jurkat-huFcγRIIIA cells after a three-week incubation at 37°C in non-heated human serum (Fig. S1).

The binding activity of HER2bsFab was investigated by flow cytometry on HER2^high^ breast cancer cells (SK-BR-3 and BT 474) and human FcγRIIIA-transfected Jurkat cells (Fig. 1B-C). HER2bsFab efficiently bound all cell lines as more than 90% of cells were positively stained. Worth mentioning, no binding was detected on the non-tumorigenic mammary epithelial cell line, hTERT-HME1, which expresses a basal level of HER2 (Fig. 1B, right panel). Relative to trastuzumab, HER2bsFab displayed a lower apparent affinity (80.1 +/− 13 nM vs. 6.8 +/− 0.6 nM) for HER2 partly explained by its monovalency. Binding of HER2bsFab was determined on all human FcγR using CHO cells expressing FLAG-tagged FcγR (Table 1). Expression levels of FcγR were checked by labeling cells with anti-FLAG. Unlike trastuzumab which bound to all FcγR except FcγRIIC, HER2bsFab bound only to FcγRIIIA and B. Regarding FcγRIIIA, trastuzumab binding was variable according to the FcγRIIIA-158 allotypes, with a 10-fold lower affinity for FcγRIIIA-158 F/F while HER2bsFab exhibited similar and high apparent affinities for both FcγRIIIA-158 allotypes. The dual specific binding of HER2bsFab to HER2 and FcγRIIIA was demonstrated by its capacity to induce, *in vitro*, the formation of rosettes of Jurkat-huFcγRIIIA cells around HER2-positive SK-OV-3 cells (Fig. 1D). No rosette forming cells were observed when an irrelevant bsFab targeting FcγRIII but not HER2 was used. Furthermore, consistent with the targeting of an original FcγRIIIA epitope [31], we confirmed that, contrary to trastuzumab, HER2bsFab did not compete with endogenous serum IgG for binding to FcγRIIIA (Fig. 1E), strongly suggesting that HER2bsFab must escape competition with serum IgG *in vivo*.

**Table 1:** Apparent affinities of HER2bsFab and trastuzumab for human Fcγ receptors

		K_D_ of trastuzumab (nM)	K_D_ of HER2bsFab (nM)
Human	FcγRI	16.3 +/− 5.8	nd
	FcγRIIA-131 H	235 +/− 7.1	nd
	FcγRIIA-131 R	258.4 +/− 12.6	nd
	FcγRIIB	66.7 +/− 4.2	nd
	FcγRIIC	nd	nd
	FcγRIIIA-158 F	194.3 +/− 1.5	10.3 +/− 0.6
	FcγRIIIA-158 V	20.4 +/− 4.4	8.7 +/− 0.1
	FcγRIIIB / NA1	330 +/− 15.2	12.3 +/− 0.2
	FcγRIIIB / NA2	276.6 +/− 1.2	8.2 +/− 0.6
	FcγRIIIB / SH	144.4 +/− 9.3	7.7 +/− 1

Values with error bars represent mean ± SEM. nd: no detectable binding.

### HER2bsFab does not inhibit cell growth

**Figure 2:**
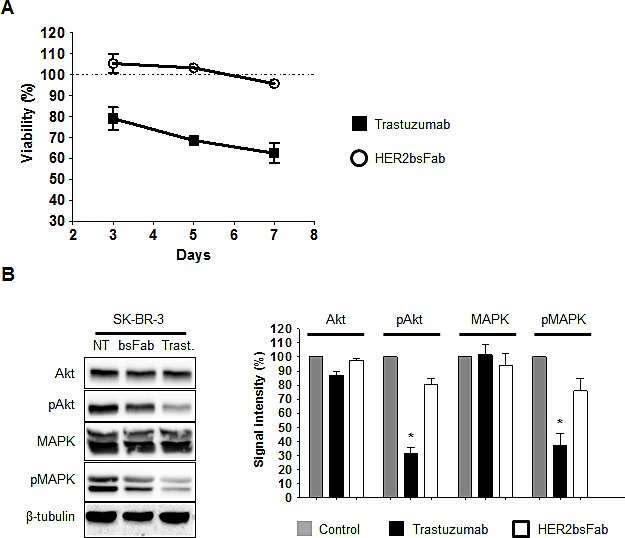
Anti-proliferative effects mediated by trastuzumab and HER2bsFab A) SK-BR-3 cells growth was followed for 3, 5 and 7 days in the presence of trastuzumab or HER2bsFab (500 nM). Cell viability was quantified using CellTiter Glo viability assay. Results are shown as percentage of control cell proliferation and expressed as mean ± SEM. All procedures were done in triplicate. B) Immunoblots evaluating the ability of HER2bsFab and trastuzumab (200 nM) to inhibit MAPK and Akt signalling pathways in SK-BR-3 cells (incubation time: 8 h). Medium alone was used as a control. The experiments were performed three times. NT: non treated; Trast: trastuzumab; bsFab: HER2bsFab. Immunoblots were quantified using the NIH Image J software after correction for β-tubulin. Values are expressed as percentage of non treated cells signal. Values with error bars represent mean ± SEM. Data were analyzed by Student's t-test. * *P* < 0.05 vs. non treated controls.

Anti-proliferative effects of HER2bsFab were determined by following SK-BR-3 cell growth for 3, 5 and 7 days in the presence of HER2bsFab or trastuzumab. No inhibitory effect of HER2bsFab was observed on SK-BR-3 cell growth while a time-dependent inhibition was achieved in the presence of trastuzumab, the maximal growth inhibition reaching about 45% after 7 days (Fig. 2A). In an attempt to explain why HER2bsFab had no anti-proliferative effect, we analyzed the impact of HER2bsFab on the activation of downstream MAPK and PI3K/AKT signaling pathways as these molecular events reflect HER2 activity (Fig. 2B). After an 8hr-incubation with either 200 nM HER2bsFab or trastuzumab, SK-BR-3 cell lysates were submitted to western blot analysis. While the levels of total MAPK and PI3K-Akt proteins were not modified, low but detectable changes in both pMAPK and pAkt levels were observed relative to untreated cells, upon HER2bsFab treatment (20-25%). As expected, a strong diminution of pMAPK and pAkt levels was observed in the presence of trastuzumab (60-70%). These results suggest that the slightly reduced activation of downstream intracellular pathways induced by HER2bsFab on SK-BR-3 cells was no sufficient to induce an inhibition of cell growth.

### HER2bsFab mediates potent NK cell cytotoxicity against HER2^high^ breast cancer cells *in vitro*

**Figure 3:**
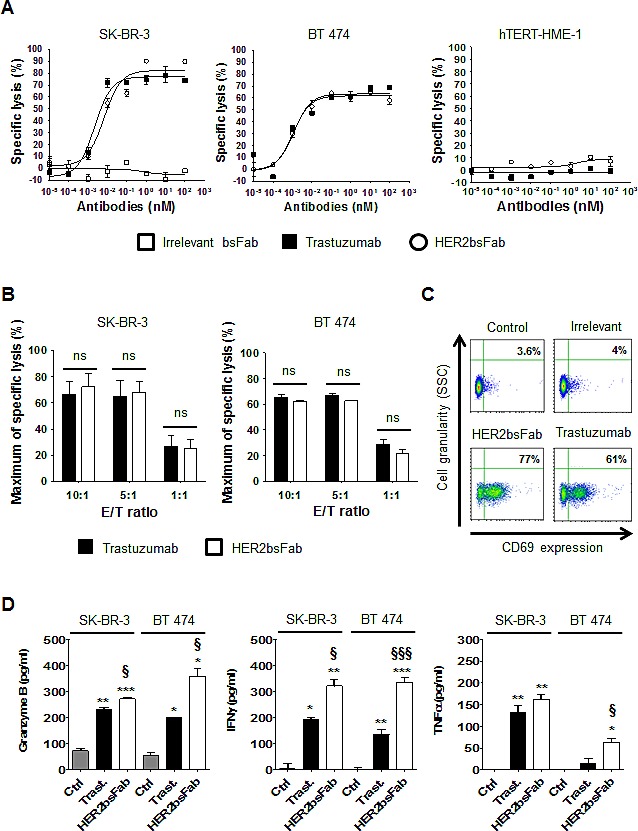
HER2bsFab mediates potent *in vitro* ADCC activity similar to trastuzumab against high-HER2-overexpressing cells A) ADCC assays were performed using human NK cells as effector cells and SK-BR-3, BT 474 or hTERT-HME-1 as target cells (E/T 10:1) in the presence of irrelevant bsFab, HER2bsFab or trastuzumab. Target cell viability was measured by CellTiter-Glo viability assay. Results are representative of at least three independent donors. Mean values ± SEM are presented. B) Impact of the E/T ratios on SK-BR-3 and BT 474 cells lysis mediated by HER2bsFab or trastuzumab (100 nM). Data are representative of two experiments performed in triplicate, and represent the mean ± SEM. ns: non significant. C) Activation status of NK cells harvested from cytotoxicity assays illustrated in A) assessed for CD69 expression, detected with APC-conjugated anti-CD69. Secretions of D) granzyme B, E) IFNγ and F) TNFα were quantified by ELISA in supernatants from cytotoxicity assays against SK-BR-3 and BT 474 cells illustrated in A). Data are representative of at least two experiments performed in triplicate, and represent the mean ± SEM. Data were analyzed by Student's t-test. * *P* < 0.05 vs. control; ** *P* < 0.01 vs. control; *** *P* < 0.001 vs. control; § *P* < 0.05 vs. trastuzumab; Ctrl: Control; Trast: trastuzumab.

The capacity of HER2bsFab to trigger tumor cell lysis by retargeting NK cells was investigated *in vitro* using a luminescent ATP-assay, as a nonradioactive alternative to the conventional Cr^51^ assay. Figure 3 shows representative results using purified unstimulated human NK cells from a healthy donor as effector cells and HER2^high^ breast cancer cells (SK-BR-3 and BT 474) or hTERT-HME1 as target cells (Fig. 3A). Very similar dose-dependent curves were obtained with trastuzumab and HER2bsFab at E/T ratio of 10:1 (Fig. 3A, left and middle panels) with comparable maximal levels of lysis (efficacy) of about 60-80% and similar EC_50_ values (potency) (2-6 pM). Since no growth inhibition was detected after overnight incubation of HER2^high^ cancer cells with saturating concentrations of trastuzumab or HER2bsFab, cell lysis was directly correlated to ADCC. Importantly, no NK cytotoxic activity was observed when an irrelevant bsFab that elicited a cytolytic response in different experimental settings [30] or when hTERT-HME-1 cells were used (Fig. 3A, right panel). Lowering the E/T ratio down to 1:1 resulted in a significant but similar reduction of lysis to about 30% both for trastuzumab and HER2bsFab (Fig. 3B), indicating that HER2bsFab remained at least as efficient as trastuzumab at low E/T ratios.

To assess that HER2bsFab-mediated lysis was correlated to FcγRIIIA engagement, the activation state of NK cells, harvested from ADCC assay against SK-BR-3 cells (E/T 10:1), was evaluated by following the expression of the activation marker CD69 by flow cytometry (Fig. 3C). No significant up-regulation of CD69 was observed in the presence of the irrelevant bsFab confirming that NK cells activation was strictly dependent on the dual engagement of both receptors. The proportion of activated NK cells was slightly higher with HER2bsFab (77%) than with trastuzumab (61%), maybe due to its higher FcγRIIIA-affinity. Secretion of granzyme B, a key cytotoxic molecule released by activated NK cells, was also quantified in supernatants harvested from ADCC assays against SK-BR-3 and BT 474 cells (Fig. 3D, left panel). Consistent with the results obtained with CD69, we found that HER2bsFab induced a granzyme B release moderately but significantly higher compared with trastuzumab. Finally, as effective antitumor immune responses are known to require the secretion of inflammatory cytokines such as IFNγ or TNFα, the ability of HER2bsFab to induce cytokines release in response to target cells was investigated (Fig. 3D, middle and right panels). Measurements of extra cellular INFγ and TNFα performed on ADCC supernatants showed that NK cells secreted higher amount of both cytokines in response to bsFab-coated SK-BR-3 and BT 474 cells compared with trastuzumab-coated cells. Taken together, these results demonstrated that, through dual engagement of HER2 and FcγRIIIA, HER2bsFab is able to strongly activate NK cells, leading to a potent and highly specific cytotoxicity toward high-HER2-overexpressing tumor cells and the secretion of pro-inflammatory cytokines.

### HER2bsFab mediates potent *in vitro* ADCC against both trastuzumab refractive and low-HER2-overexpressing breast cancer cells

We next determined whether enhanced affinity for FcγRIIIA could be translated into increased ADCC activity against trastuzumab-refractive and low-HER2-overexpressing breast cancer cell lines. To support this hypothesis, we evaluated the cytotoxic activity of HER2bsFab against two different breast cancer cell lines, MCF-7 which weakly overexpresses HER2 and JIMT-1 which, although overexpressing HER2, displayed a low trastuzumab-binding capacity due to overexpression of mucin 4 (MUC4) [32].

**Figure 4:**
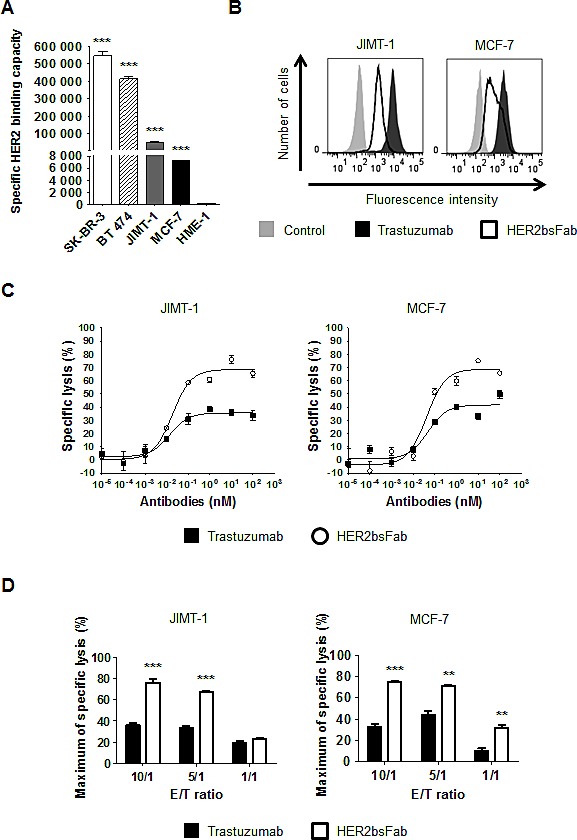
*In vitro*, HER2bsFab induces potent ADCC against low-HER2-overexpressing and trastuzumab refractive cells A) Specific HER2 binding capacities of SK-BR-3, BT 474, JIMT-1, MCF-7 and hTERT-HME-1 cells were assessed by DAKO QIFIKIT. Values with error bars represent mean ± SEM. Data were analyzed by Student's t-test. *** *P* < 0.001 vs. HME-1 cells. B) Binding of isotype control, biotinylated trastuzumab or biotinylated HER2bsFab to JIMT-1 or MCF-7 cells. Bound antibodies were detected using PE-labeled streptavidin. C) ADCC assays were performed using JIMT-1 or MCF-7 cells as target cells and human NK cells as effector cells (E/T 10:1) and HER2bsFab or trastuzumab. Target cell viability was measured by CellTiter Glo viability assay. Results are representative of at least three independent donors. Values with error bars represent mean ± SEM. D) Impact of the E/T ratios on JIMT-1 or MCF-7 cells lysis mediated by HER2bsFab or trastuzumab (100 nM) cells. Values with error bars represent mean ± SEM and are representative of two experiments performed in triplicate. Data were analyzed by Student's t-test. ** *P* < 0.01 vs. trastuzumab; *** *P* < 0.001 vs. trastuzumab.

The mean number of accessible HER2 binding sites (specific antigen binding capacity, SABC) of the different cell lines was first estimated by the Dako QIFIKIT^®^ (Fig. 4A). According to the consensual clinical classification of HER2 expression in tumors, MCF-7 cells (HER2^low^ / IHC score 1+) weakly overexpressed HER2 with a SABC (~7,260 sites/cell) significantly lower than SK-BR-3 or BT 474 cells (HER2^high^/ IHC score 3+, SABC > 400,000 sites/cell) but still significantly higher than hTERT-HME1 cells (SABC ~127 sites/cell). In accordance with the results described by Nagy *et al.* [33], and despite similar number of HER2 gene copies, the amount of accessible HER2 receptors at the surface of JIMT-1 cells (SABC ~53,800) appeared to be about 8 to 10-fold lower than that determined for the two HER2^high^ cancer cells, due to overexpression of MUC4. Consistent with these results, JIMT-1 and MCF-7 cells displayed a significantly weaker labeling with both trastuzumab and HER2bsFab compared to SK-BR-3 or BT 474 cells (Fig. 4B). Nevertheless, HER2bsFab binding activity still remained efficient on these two cell lines as more than 90% of cells were labeled in each case.

Cytotoxic assays were then performed, using purified resting human NK cells and E/T ratio of 10:1 (Fig. 4C). As expected, trastuzumab triggered low levels of ADCC against MCF-7 and JIMT-1 cells (40 and 38% respectively). By contrast, HER2bsFab demonstrated high cytotoxic activity on both cell lines, with maximum rates of specific lysis of 78% and 62% respectively. Interestingly, the higher ADCC potential of HER2bsFab against MCF-7 cells remained evident at the three E/T ratios tested (Fig. 4D, right panel). On the contrary, the difference in ADCC activity against JIMT-1 cells between the two antibodies faded at the lowest E/T ratio (Fig. 4D, left panel).

**Figure 5:**
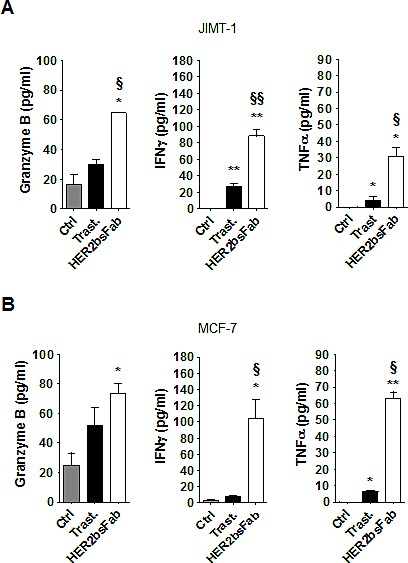
HER2bsFab efficacy against trastuzumab-refractive cells acts through an efficient activation of NK cells NK activation was assessed by evaluating secretion of granzyme B, IFNγ and TNFα in supernatants of cytotoxicity assays on A) JIMT-1 and B) MCF-7 cells by ELISA. Measurements were performed in triplicate. Values with error bars represent mean ± SEM. Data were analyzed by Student's t-test. * *P* < 0.05 vs. control; ** *P* < 0.01 vs. control; § *P* < 0.05 vs. trastuzumab; §§ *P* < 0.01 vs. trastuzumab.

We subsequently measured the release of granzyme B and pro-inflammatory cytokines in the supernatants harvested from above ADCC assays (Fig. 5). As could be expected from the decrease of trastuzumab efficiency, the amounts of granzyme B and pro-inflammatory cytokines released during trastuzumab-driven ADCC against both MCF-7 and JIMT-1 cells were rather low, not always reaching significance over spontaneous release. Likewise, granzyme release and cytokine secretion by NK cells were also impaired in response to HER2bsFab-coated cells, albeit to a lesser extent. This finding, in line with those obtained with high-HER2-overexpressing cells, supported the idea that HER2bsFab is more potent than trastuzumab for inducing NK cell activation whatever the level of HER2 expression.

### HER2bsFab mediates *in vivo* antitumor responses against both HER2^high^ and trastuzumab-refractive tumors

Before investigating the therapeutic activity of the two antibodies in mouse models, their binding to murine FcγR was checked by flow cytometry on stably transfected CHO cells expressing FLAG-tagged murine FcγR (Table 2). As expected, trastuzumab bound all four murine activating and inhibiting FcγR, with apparent affinities ranging from 46 to 309 nM, while HER2bsFab only recognized FcγRIII and FcγRIV (K_D_ ~ 20 nM). These results were consistent with those on immune effector cells collected from spleen of nude mice demonstrating that both antibodies were able to bind on murine macrophages, monocytes, NK cells and neutrophils (Fig. S2).

**Table:** Table 2:Apparent affinities of HER2bsFab and trastuzumab for mouse Fcγ receptors

	K^D^ of trastuzumab (nM)	K^D^ of HER2bsFab (nM)
FcγRI	110.5 +/− 11.8	nd
FcγRIIB	309.1 +/− 27.9	nd
FcγRIII	198.2 +/− 4.5	17.2 +/− 0.58
FcγRIV	46.3 +/− 1.2	26.4 +/− 2

Values with error bars represent mean ± SEM. nd: no detectable binding.

Knowing that HER2bsFab interacts with mouse resident immune cells, HER2bsFab *in vivo* activity was examined using HER2^high^ (BT 474), HER2^low^ (MCF-7) and trastuzumab-refractive (JIMT-1) xenograft models. BT 474 cells were chosen as SK-BR-3 cells have been reported to poorly form xenograft in athymic mice [34]. Athymic NMRI Nude mice were injected with 10^7^ cells subcutaneously and treatments were started once tumors reached about 180-250 mm^3^. Irrelevant bsFab and HER2bsFab were injected i.p. at 5 mg/kg three times per week while trastuzumab was injected at the same dose two times per week. In all xenograft models, a 100% tumor-take was reached and no adverse effects on body weight or general behavior were observed for all treated mice.

**Figure 6:**
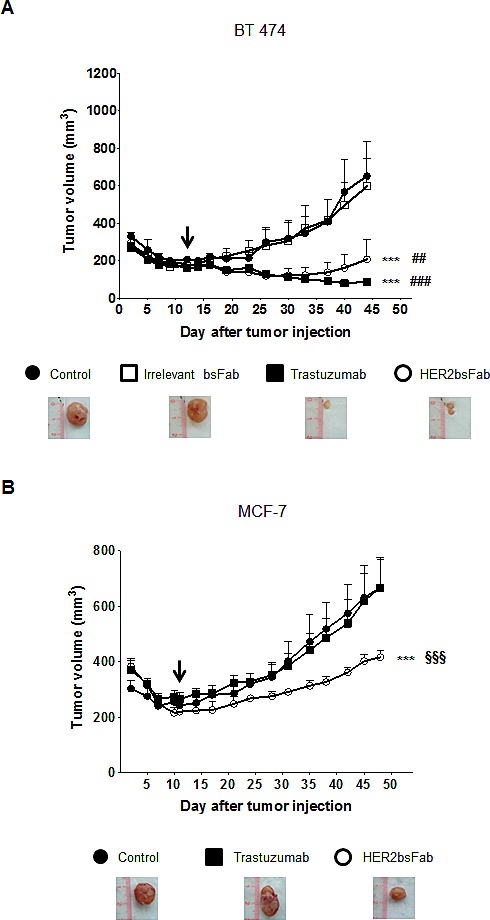
*In vivo* anti-tumor activity of HER2bsFab All tumors were xenografted subcutaneously in NMRI Nude mice. Tumor volumes of A) BT 474 or B) MCF-7 tumors were measured after treatment with irrelevant bsFab (5 mg/kg), HER2bsFab (5 mg/kg), PBS (control buffer) or trastuzumab (5 mg/kg). Values with error bars represent mean ± SEM. Data were analyzed by One way ANOVA test. *** *P* < 0.001 vs. control buffer group; ### *P* < 0.001 vs. irrelevant bsFab group; ## *P* < 0.01 vs. irrelevant bsFab group; §§§ *P* < 0.001 vs. trastuzumab group. Beginnings of treatments were illustrated by black arrows. Representative images of tumors treated with negative controls, trastuzumab or HER2bsFab are illustrated for each model.

As shown in Figure 6A, HER2bsFab displayed a potent anti-tumor activity comparable to that of trastuzumab against BT 474 cells, as evidenced by the statistically different size of tumors between controls (PBS and irrelevant bsFab) and treated groups. Importantly, no significant difference was observed between control buffer and irrelevant bsFab suggesting that the antitumor response produced by HER2bsFab is specific to HER2 recognition. Regarding the JIMT-1 xenograft model, trastuzumab moderately but significantly delayed JIMT-1 tumor progression compared with the non-treated group (Fig. S3). HER2bsFab also tended to delay tumoral growth without, however, reaching a significant benefit over the control group. By contrast, HER2bsFab treatment significantly reduced MCF-7 tumor growth in comparison to PBS while no tumor growth delay was observed in tumor-bearing mice treated with trastuzumab (Fig. 6C). Altogether, these data demonstrated that, relative to trastuzumab, HER2bsFab displayed significant benefit for treating both *in vitro* and *in vivo* tumors overexpressing only weakly HER2.

### FcγRIIIA-158 F/V polymorphism has no incidence on *in vitro* cytotoxic activity of HER2bsFab against HER2^low^ tumor cells

**Figure 7:**
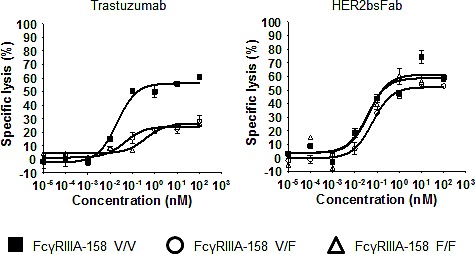
Effect of FcγRIIIA polymorphism on the ADCC triggered by HER2bsFab ADCC assays were performed using human FcγRIIIA-158 V/V, F/V or F/F NK cells as effector cells and MCF-7 as target cells (E/T 5:1) in the presence of various concentrations of trastuzumab or HER2bsFab. All experiments were performed two times in triplicate. Values with error bars represent mean ± SEM.

As these promising pre-clinical results suggested potential clinical interest in extending the proportion of treatable HER2 breast cancers, one important issue to look at was the absence of influence of the FcγRIIIA-158 F/V polymorphism in the case of tumors overexpressing only weakly HER2. Indeed, it has been shown that the FcγRIIIA-158 F/V and F/F allotypes, present in more than 80% of the caucasian population [13], are associated with worse clinical response upon trastuzumab treatment. In this respect, we showed that HER2bsFab-triggered ADCC against HER2^low^ MCF-7 cells using NK cells from FcγRIIIA-158 V/V, F/F or V/F donors (E/T 5:1) was not affected by the FcγRIIIA-158 polymorphism as EC_50_ values ranged from 44 to 56 pM and maximal lysis, from 52 to 57% whatever the FcγRIIIA-158 allotype (Fig. 7). By contrast, as expected, both the potency and the efficacy of trastuzumab were significantly modulated according to FcγRIIIA-158 allotype.

## DISCUSSION

Trastuzumab represents the archetype of clinically effective targeted immunotherapy of solid tumors, as it has contributed to real improvements in the outcome of metastatic breast cancer strongly overexpressing HER2. However, significant subsets of patients do not benefit from trastuzumab treatment due either to primary resistance, to relapse during the course of treatment due to acquired resistance or to low HER2 IHC score [5, 6]. Besides the limitations related to its own characteristics, trastuzumab also suffers from the disadvantages of IgG antibodies, namely FcγRIIIA-158 polymorphism of NK cells [13], competition with endogenous IgG [35], binding on inhibitory receptor FcγRIIB [36] and Fc fucosylation issues [37]. Thus, considerable efforts are currently made to optimize the effector function of trastuzumab mainly through removal of fucose glycosylation [15] or through mutations to overcome the polymorphism issues [16, 38]. However, although improving FcγRIIIA binding in a significant way, these strategies do not fully bypass the limitation of inhibitory FcγRIIB binding [16].

Another promising strategy is the development of bispecific antibodies able to recruit and activate various immune effector cells at the tumor site. Several anti-HER2 bispecific antibodies targeting neutrophils, monocytes, and macrophages via FcγRI [39], T-cells via CD3/CD28 [40-42] or NK cells via FcγRIIIA [43, 44] have been designed. However, despite encouraging *in vitro* and preclinical results, only one bispecific antibody targeting CD3 [42] is under clinical trial, highlighting the need to improve further the design of these molecules. Using our versatile Fab-like bispecific antibody format [30] based on the use of llama single domain antibodies, we developed a bispecific antibody, named HER2bsFab, that targets human HER2 antigen and activating FcγRIIIA receptor.

Consistent with our previous studies [30], HER2bsFab was well produced in *E. coli* and highly stable due to the absence of linker. Compared to optimized Fc formats, HER2bsFab displays unique and interesting features. It is selective for FcγRIII as no binding to FcγRI, FcγRIIA or inhibitory receptor FcγRIIB was observed. Moreover, it binds the FcγRIIIA receptor with an apparent affinity 36-fold higher than trastuzumab, targeting an epitope outside the Fcγ binding site [31], thus bypassing competition with endogenous IgG. HER2bsFab specifically binds to an epitope of the extracellular domain of HER2 that is different from that of trastuzumab [45], with an intermediate affinity (60 nM). This feature might be an advantage over trastuzumab as several studies have described the existence of a binding site barrier that limits tumor penetration of high affinity antibodies [46, 47]. As well, Cao *et al.* [48] recently reported that high affinity scFv antibody-based immunotoxins induced more hepatotoxicity than their low affinity counterparts, due to an important formation of immune complexes with soluble shed tumor antigen. An intermediate affinity for the well known shed extracellular domain of HER2 could avoid such side effects.

Exploration of *in vitro* direct HER2-driven properties of HER2bsFab showed that, unlike trastuzumab, HER2bsFab had no anti-proliferative activity on HER2^high^ SK-BR-3 cell, despite a minor effect on downstream MAPK and PI3K/Akt signaling pathways. This result could be explained by either the epitope specificity of HER2bsFab, its monovalency and/or its intermediate affinity for HER2 as we demonstrated that the same anti-HER2 sdAb produced in a bivalent format (Fc fusion, apparent K_D_ of 2 nM), showed an anti-proliferative effect (30%) at high concentration. As ADCC and signaling effects occur independently [49], the absence of anti-proliferative effects of HER2bsFab should not be detrimental for *in vivo* anti-tumor activity and could avoid the development of resistance related to upregulation of alternative signaling pathways and/or to altered intracellular signaling observed upon long-term exposure to trastuzumab [50, 51].

We showed that HER2bsFab-mediated ADCC against HER2^high^ breast cancer cells was similar to that of trastuzumab both in terms of efficacy (maximal lysis) and potency (EC_50_), even at low E/T ratio (1:1). Worth mentioning, HER2bsFab retained its high ADCC activity at picomolar concentration using unstimulated human PBMC as effectors (E/T: 25/1, Fig. S4). The presence of neutrophils *in vitro* did not appear to modify the killing efficacy of HER2bsFab, in agreement with previously published data [52] (Fig. S5). The analysis of ADCC pathway showed that, through FcγRIIIA engagement, HER2bsFab, as trastuzumab, induces a perforin-mediated cytotoxicity, stimulating NK cell degranulation and cytokine secretion in response to target cell recognition. Fauriat *et al* [53] have shown that different thresholds exist in activating signals to induce NK cell effector responses: chemokine secretion requires the lowest activating signals, moderate ones induce degranulation and the strongest ones induce cytokine secretion. Interestingly, relative to trastuzumab, HER2bsFab elicited a higher level of secreted IFNγ suggesting that HER2bsFab induces a stronger NK activation signal. This finding was consistent with the slightly greater percentage of CD69 positive NK cells and the much higher level of granzyme B, further emphasizing the substantial difference in FcγRIIIA engagement between HER2bsFab and trastuzumab, likely due to more stable crosslinking of FcγRIIIA by HER2bsFab. However, despite the higher NK activation mediated by HER2bsFab, no difference in maximum lysis was observed compared with trastuzumab against both SK-BR-3 and BT 474 cells. One explanation could be that the high HER2 density displayed by these cell lines shades off the differences in affinity for both HER2 and FcγRIIIA between the two antibodies, the bivalence of trastuzumab against HER2 compensating its low affinity for FcγRIIIA.

The more potent effector functions mediated by HER2bsFab raise the hypothesis that HER2bsFab could potentially be beneficial in a context of trastuzumab-refractive and low-HER2-overexpressing breast cancers. To address this question, we analyzed the *in vitro* efficacy of HER2bsFab against two models of trastuzumab refractive breast cancers: MCF-7, an HER2 non-amplified cell line not eligible to trastuzumab treatment due to a weak HER2 overexpression, and JIMT-1, a model of HER2-overexpressing, trastuzumab resistant cell line. Several mechanisms are likely underlying JIMT-1 resistance among which (i) a partial masking of HER2 by overexpressed MUC4 [33] and (ii) the absence of HER2-driven effects of trastuzumab [54]. The absence of anti-proliferative effects of either trastuzumab or HER2bsFab on these two cell lines allowed a direct comparison of their ADCC capacity.

For sake of comparison between cell lines and because heterogeneous data are found in the literature regarding the HER2 status of MCF-7, maybe due to biological differences among MCF-7 cell lines [55], the HER2 binding capacity of all cell lines tested was assessed. We thus confirmed that MCF-7 cells displayed a low but significant HER2 overexpression (SABC 60-fold higher than that of hTERT-HME-1 cells) despite an absence of gene amplification. We show in this study that *in vitro*, HER2bsFab retained its full killing efficacy against both JIMT-1 and MCF-7 cells at high E/T ratio (10/1) independently of HER2 status while trastuzumab-mediated ADCC proved to be much less efficient than against HER2^high^ cells. NK cell activation mediated by HER2bsFab upon trastuzumab-refractive cell engagement still remained stronger than that mediated by trastuzumab as demonstrated by higher secretion levels of granzyme B and pro-inflammatory cytokines. Taken together, these results suggest that comparatively to trastuzumab, the threshold of HER2 density on target cells required for inducing HER2bsFab-mediated NK cell activation and ADCC was lower, maybe due to its enhanced FcγRIIIA binding.

Interestingly, at low E/T ratio (1:1), a condition closer to the situation at the tumor site, HER2bsFab still remained significantly more potent than trastuzumab for inducing MCF-7 lysis, underlying the potential benefit of HER2bsFab against tumors expressing low HER2 levels *in vivo*.

Taking benefit from the fact that HER2bsFab can bind mouse resident immune cells including NK cells, macrophages and neutrophils, we demonstrated that HER2bsFab triggers a potent ADCC activity *in vivo*. In NMRI Nude mice with established HER2^high^ BT 474 xenograft, HER2bsFab displayed the same potential as trastuzumab at inhibiting tumor growth. In the absence of anti-proliferative properties, the HER2bsFab-mediated effects on tumor growth can exclusively be attributed to ADCC and these effects are target-restricted since an irrelevant bsFab had no impact on tumor growth. Although the toxicity of the HER2bsFab remains to be further evaluated, it must be pointed out that, in all *in vivo* studies, no apparent detrimental off target toxicity was observed as monitored by animal survival and weight curves despite repeated injections. Along this line, we showed that, *in vitro*, HER2bsFab was unable to elicit ADCC against the non-tumorigenic human mammary epithelial cells (hTERT-HME1).

In a context of low-HER2-overexpressing and trastuzumab-refractive tumors, the potency of bsFab was tested in NMRI Nude mice with established MCF-7 or JIMT-1 xenografts. Consistent with several studies showing the ability of trastuzumab to inhibit outgrowth of detectable JIMT-1 tumors [54, 56], a moderate growth inhibition was observed with trastuzumab. Unexpectedly, while a stronger inhibitory effect of bsFab could have been expected based on *in vitro* ADCC assays, HER2bsFab reduced JIMT-1 tumor growth, closely approaching significance (P=0.057). This finding could be related to the observation that the difference in ADCC activity between the two antibodies was abrogated, *in vitro*, at low E/T ratio (1:1) with this cell line. In contrast, a more striking difference was observed on established MCF-7 xenografts. Unlike trastuzumab that, as described in the literature, had no effect against MCF-7 tumors, HER2bsFab induced a significant slowdown of tumor growth suggesting that HER2bsFab could provide a clinical benefit in the case of both high- and low-HER2-overexpressing breast cancers.

Our results are in line with recent reports on the benefit of enhancing trastuzumab affinity for FcγRIIIA on its antitumor activity *in vivo* [15, 16] and emphasize the role of ADCC as mechanism of action in the *in vivo* antitumor effects of ADCC-promoting antibodies in solid tumors. Moreover, by targeting an epitope on FcγRIIIA distant from the Fc binding site, HER2bsFab completely bypasses the FcγRIIIA-158 polymorphism issue that restricts the therapeutic efficiency of trastuzumab [13] for all FcγRIIIA-158 F carriers. HER2bsFab could thus represent an interesting approach for an efficient HER2-targeting treatment independent of both patient FcγRIIIA-158 phenotype and HER2 status. Recently, clinical evidences have been reported on the benefit of multiple HER2-targeted therapies [57, 58]. In this regard, the different epitope specificities of HER2bsFab for both HER2 and FcγRIIIA compared with trastuzumab and pertuzumab [31, 45] could be of clinical interest for combinatorial therapies, as no interference in binding and killing efficacy should be expected. Finally, in addition to patients with low HER2 IHC score, patients experiencing tumor progression during trastuzumab-based treatment may also benefit from HER2bsFab.

In conclusion, we have designed a bispecific antibody displaying a moderate affinity for the tumor-targeted antigen and a unique, specific and high FcγRIII-binding affinity using ADCC as major mechanism of action. Endowed with a high potential to inhibit HER2^high^ tumor growth, HER2bsFab also overcomes *in vitro* and *in vivo* limitations associated with the threshold of HER2 expression level on target cells and with NK cell polymorphism for eliciting efficient anti-tumor activity. This study raises the perspective to enable the treatment of a broader population of patients than that eligible with current HER2-targeted therapies.

## MATERIALS AND METHODS

### Construction and production of HER2bsFab

The anti-HER2 × FcγRIII bispecific antibody was constructed by cloning cDNA of the anti-HER2 sdAb (HER2.C7b) [59] into the expression vector pBAT14 upstream the anti-FcγRIII sdAb (CD16.21) cDNA [30]. The resulting bispecific antibody, named HER2bsFab, was produced in *E.coli* (*DH5α* strain) periplasm and purified as previously described [30]. Endotoxin concentration was checked using the Pierce LAL Chromogenic Endotoxin Quantitation Kit (Thermo Scientific). Commercial trastuzumab (Herceptin^®^, Roche) was a kind gift from TrGET platform (U1068 INSERM, Marseille, France).

### Effector cells and target cell lines

Breast cancer cell lines SK-OV-3 (ATCC HTB-77), SK-BR-3 (ATCC HTB-30), BT-474 (ATCC HTB-20), MCF-7 (ATCC HTB-22) and the immortalized epithelial cell line hTERT-HME-1 (ATCC CRL-401) were purchased from ATCC and JIMT-1 (ACC 589) cell line from DSMZ. Human FcγRIIIA transfected Jurkat lymphoma T cells (Jurkat-huFcγRIIIA cells) were a gift of Pr. Eric Vivier (Marseille, France) and were not authenticated. Stably transfected CHO cells expressing FLAG-tagged human FcγRI (CNCM I-4383), FcγRIIA-131 H (CNCM I-4384), FcγRIIA-131 R (CNCM I-4385), FcγRIIB (CNCM I-4386), FcγRIIC (CNCM I-4387), FcγRIIIA-158 F/F (CNCM I-4388), FcγRIIIA-158 V/V (CNCM I-4389), FcγRIIIB/NA1 (CNCM I-4390), FcγRIIIB/NA2 (CNCM I-4391), FcγRIIIB/SH (CNCM I-4392) were provided by Collection Nationale de Cultures de Microorganismes (CNCM, Institut Pasteur, France) [60]. Stably transfected CHO cells expressing FLAG-tagged mouse FcγRs were reported before [61]. Human peripheral blood mononuclear cells (PBMCs) were isolated from fresh peripheral blood of healthy donors (Etablissement Français du Sang (EFS), Marseille, France) by Ficoll LSM 1077 (PAA) gradient centrifugation. All donors were of FcγRIIIA-158 F/F or F/V phenotype unless otherwise stated. NK cells were isolated as previously described [30]. Frozen PBMCs from FcγRIIIA-158 V/V, F/F or V/F donors were kindly provided by Dr. Christophe Picard (EFS, Marseille, France).

### Characterization of HER2bsFab by fluorescence cytometry

HER2 binding capacity of tumor cell lines was quantified by DAKO QIFIKIT (DAKO Cytomation), according to the manufacturer's protocol using mAb ER-23 (Santa Cruz) as primary antibody. HER2 quantity was expressed as specific antibody-binding capacity units after subtraction of isotype control (mouse IgG2b) background. For binding experiments, HER2bsFab and trastuzumab were biotinylated *in vitro* using Ez-link micro NMHS-PEO4-biotinylation kit (Perbio science). The impact of biotinylation on antigen binding was checked by comparing binding activities of biotinylated antibodies and their unlabeled counterparts by flow cytometry. Binding and apparent affinities were determined by flow cytometry as previously described [59] using 2×10^5^ Jurkat-huFcγRIIIA, SK-BR-3, stably transfected CHO cells expressing FLAG-tagged human or mouse FcγRs and biotinylated antibodies (0.05 nM to 2000 nM). Stability assays in human serum were performed as previously described [30] for 21 days with biotinylated antibodies. Competition assays with endogenous IgGs were performed by incubating Jurkat-huFcγRIIIA cells in the presence of human serum (20 or 100%) and sub-saturating concentration of biotinylated HER2bsFab (50 nM) or trastuzumab (200 nM). Bound antibodies were detected by flow cytometry using PE-labeled streptavidin [30].

### Rosette forming cell assay

Rosette forming cell assays were performed as previously described [30]. SK-OV-3 cells (5×10^6^) were labeled for 10 min at 37°C with 20 μM CellTrace™ CFSE (Invitrogen) in PBS/0.1% BSA and Jurkat-huFcγRIIIA cells (5×10^6^) for 30 min at 37°C with 100 μM CellTracker™ Red CMTPX (Invitrogen).

### *In vitro* viability assays

Target cells (5×10^3^ cells) were seeded on 96-well plates, incubated overnight for cell attachment and treated or not with increasing concentrations (0.05 to 500 nM) of trastuzumab or HER2bsFab. After 3, 5 and 7 days, cell viability was quantified with CellTiter-Glo Luminescent Cell Viability Assay according to manufacturer's protocol (Promega). Percent viability was calculated as follows: (T_Ab_ × 100) / T with T = Non-treated target cells luminescent signal, and T_Ab_ = Target cells + Antibody luminescent signal. All procedures were done in triplicate.

### MAPK and Akt activation

After an 8hr-incubation in the absence or presence of 200 nM trastuzumab or HER2bsFab, SK-BR-3 cell lysates (25 μg) were separated by SDS-PAGE under reducing conditions. Western blots were probed with anti-Akt (11E7, 1/1000), anti-pAkt (D9E, 1/2000), anti-MAPK (137F5, 1/1000) and anti-pMAPK (20G11, 1/1000) antibodies (Cell Signaling Technology) and anti-rabbit (sc-2004, 1/5000, Santa Cruz Biotechnology) or anti-mouse (A2304, 1/5000, Sigma-Aldrich) horseradish peroxidase (HRP)-conjugate secondary antibodies (sc-2004, Santa Cruz Biotechnology) for detection. β-*tubulin detected with anti-tubulin (TUB 2.1, 1/4000, Sigma Aldrich)* was used as protein *loading* control.

### *In vitro* ADCC assays

Assays were performed as previously described [30]. Target cell viability was quantified using CellTiter-Glo Luminescent Cell Viability Assay. Percent cytotoxicity was calculated as follows: [T - (T_EAb_ - E)] / [(T - (T_dead_ - E)] × 100 with T = Target luminescent signal, E = effector luminescent signal, T_dead_ = luminescent signal of target cells lysed with 1% Triton X100 solution and T_EAb_ = Target + Effector + Antibody luminescent signal.

### NK activation assays

Supernatants were harvested from ADCC assays and frozen at ×20°C. Secreted human IFNγ and TNFα were measured by ELISA using the READY-SET-GO human IFNγ or TNFα kits as described by the manufacturer (eBioscience). Secreted human granzyme B was measured by ELISA using the Human Granzyme B Platinum ELISA kit (eBioscience). Cell surface expression of CD69 on harvested NK cells was analyzed by flow cytometry after labeling with an APC-conjugated anti-CD69 IgG (Miltenyi).

### Tumor growth studies

NMRI Nude mice were subcutaneously injected with 10^7^ cells in a 1/2 (v/v) Matrigel/PBS suspension (BD Biosciences). When tumors reached an average of 180-250 mm^3^, mice were randomly divided into treatment groups (n=6). HER2bsFab (5 mg/kg) and negative controls (saline buffer or irrelevant bsFab targeted to mesothelin (5 mg/kg)) were given intraperitoneally (i.p.) three times per week and trastuzumab (5 mg/kg) i.p. twice weekly. All experiments were performed in agreement with the French Animal Protection Law with the permission of local authorities

### Statistical analysis

All data are presented as mean ± SEM. Statistical analysis of *in vitro* data was performed by Student's unpaired t. *In vivo* data were analyzed by One-Way ANOVA test. Values of P < 0.05 were considered significant.

Material and Methods are further detailed in the supplementary material.

## SUPPLEMENTAL MATERIALS AND FIGURES


