# Supplementary material for: A FcγRIII-engaging bispecific antibody expands the range of HER2-expressing breast tumors eligible to antibody therapy
Source: Oncotarget. 2014 Jun 11;5(14):5304–19. doi: 10.18632/oncotarget.2093 (PMC4170649; doi:10.18632/oncotarget.2093)
Supplement: Supplementary file 1 [file oncotarget-05-5304-s001.pdf]

A FcγRIII-engaging bispecific antibody expands the range of HER2-expressing breast tumors eligible to antibody therapy

SUPPLEMENTAL MATERIALS

Effector cells and target cell lines. All experiments were performed with recently thawed vials. SK-BR-3, BT 474 and Jurkat-huFcγRIIIA cells were cultured in RPMI 1640+ Glutamax-I (Invitrogen) containing 10% (v/v) FBS (PAA) and 0.5 mg/ml geneticin (Invitrogen) for Jurkat-huFcγRIIIA cells. JIMT-1 and MCF-7 cells were cultured in DMEM + Glutamax-I (Invitrogen) complemented with 10% (v/v) FBS and HME-1 cells in MEGM medium (Lonza). Stably transfected CHO cells expressing FLAG-tagged **human and mouse FcγR** were cultured in RPMI 1640+ Glutamax-I (Invitrogen) containing 10% (v/v) FBS, 1% (v/v) non essential amino acids (Invitrogen), 1% (v/v) sodium pyruvate (Invitrogen), **0.5 mg/mL geneticin and, for CHO expressing-FLAG-tagged human FcγRI and FcγRIIIA, 0.25 mg/mL zeocin** (Invitrogen) and 0.5 mg/mL geneticin. Stably transfected CHO cells expressing mouse FcγRII were cultured in RPMI 1640+ Glutamax-I (Invitrogen) containing 10% (v/v) FBS, 1% (v/v) non essential amino acids (Invitrogen), 1% (v/v) sodium pyruvate (Invitrogen) and 0.5 mg/ml geneticin. Untouched NK cells were isolated as previously described [1] after overnight incubation of PBMC (10^6/mL) at 37°C in RPMI 1640 + Glutamax-I medium supplemented with 10% FBS. Human neutrophils were isolated from freshly collected blood of healthy donors as described in [2].

MAPK and Akt activation. SK-BR-3 cells were cultured in serum-containing medium ($5x10^5$ cells per well) at 37°C until 80% of confluence was reached. The medium was replaced by serum-free medium containing 200 nM Trastuzumab or HER2bsFab and culture was prolonged for 8 hours at 37°C. Cells were resuspended in lysis buffer (50 mM Hepes, 150 mM NaCl, 1 mM EDTA, 1 mM EGTA, 10% glycerol, 1% Triton X-100, 25 mM NaF, 10 µM ZnCl2, 1 mM Na3VO4, phenylmethylsulfonylfluoride (PMSF) and protease inhibitors cocktail (Sigma-Aldrich)) and after estimation of protein concentration using DC Protein Assay (Biorad), cell lysates (25 µg) were separated by SDS-PAGE under reducing conditions. Immunoblots were developed using Immobilon Western Chemiluminescent HRP Substrate (Millipore). Chemiluminescence was detected using Fusion FX7 device (Fisher Bioblock Scientific, Illkirch, France). Experiments were performed three times. Gels were quantified and analyzed with the NIH Image J software and are corrected for β-tubulin.

In vitro ADCC assays. NK cell mediated ADCC assays were performed as previously described [1]. Briefly, unstimulated human NK cells were incubated overnight (12hr) with target cells at various E/T ratios and various concentrations (0.01 pM to 100 nM) of either bsFabs (HER2bsFab or irrelevant bsFab) or Trastuzumab. NK cells co-incubated with target cells alone were used as controls. Target cell viability was quantified CellTiter-Glo Luminescent Cell Viability Assay. Spontaneous NK cytotoxicity was always below 10% independently of the E/T ratio. PBMC-mediated ADCC assays were performed using BT 474 cells as target cells (E/T: 25/1) for 4hrs and cytotoxicity was determined using LDH assay (Roche). Percent cytotoxicity was calculated as follows: $[T - (T_{EAb} - E)] / [(T - (T_{dead} - E)] \times 100$ with T = Target luminescent signal, E = effector luminescent signal, T_{dead} = luminescent signal of target cells lysed with 1% triton solution and T_{EAb} = Target + Effector + Antibody luminescent signal. All assays were done in triplicate with at least 3 different healthy donors.

Impact of unactivated neutrophils on ADCC. Briefly, unstimulated human NK cells were incubated overnight (12hr) with target cells E/T : 10/1 with 10nM trastuzumab or HER2bsFab in the presence or absence of unactivated neutrophils (neutrophil/NK: 10/1). No significant lysis was observed when neutrophils alone were co-incubated with target cells (E/T: 100/1) and 10nM trastuzumab or HER2bsFab.

Binding assay on immune cells from mouse spleen. Spleens were collected from two NMRI Nude mice (Janvier Laboratories, France). Splenic cells were obtained from whole spleens by teasing across a sterile nylon membrane and red blood cells were lysed 10 min in ACK lysis buffer (Invitrogen). After a washing step in RPMI medium, cells were plated ($2x10^5$ cells by well) in a 96-well plate. Bindings were determined by flow cytometry using biotinylated antibodies (200 nM) and detected using PE-labeled streptavidin. Mouse macrophages and mococytes were stained with FITC-labeled anti-CD11b. Mouse NK cells were stained using APC-labeled anti-CD49b antibody.

Tumor growth studies. For all xenograft models, NMRI Nude female mice (4 weeks-old) (Janvier Laboratories, France) were injected with 10^7 cells in a 300 µL 1/2 Matrigel/PBS suspension (BD Matrigel, BD Biosciences) subcutaneously in the right flank. For BT-474 and MCF-7 xenografts, a 60 day-release estrogen pellet (0.72mg 17β-estradiol; Innovative Research of America) was implanted 2 days before cell inoculation. When tumors reached an average of 180-250 mm^3, mice were randomly grouped (n=6) into treatment cohorts. HER2bsFab (5 mg/kg) and negative controls (saline buffer or irrelevant bsFab targeted to mesothelin (5 mg/kg)) were given intraperitoneally (i.p.) three times per week and trastuzumab (5 mg/kg) twice weekly. Tumors were measured twice a week using a Vernier caliper and tumor volume was calculated as $V = (L \times W^2)/2$ where L and W represent length and width, respectively. At the end of experiment, mice were sacrificed in accordance with institutional guidelines. All experiments were performed in agreement with the French Animal Protection Law with the permission of local authorities. All tumor growth data were analyzed using the GraphPad Prism software (GraphPad software).

1. Rozan C, Cornillon A, Petiard C, Chartier M, Behar G, Boix C, Kerfelec B, Robert B, Pelegrin A, Chames P, Teillaud JL and Baty D. Single-domain antibody-based and linker-free bispecific antibodies targeting FcgammaRIII induce potent antitumor activity without recruiting regulatory T cells. Mol Cancer Ther. 2013; 12(8):1481-1491.
2. Oh H, Siano B and Diamond S. Neutrophil isolation protocol. Journal of visualized experiments : JoVE. 2008; (17).

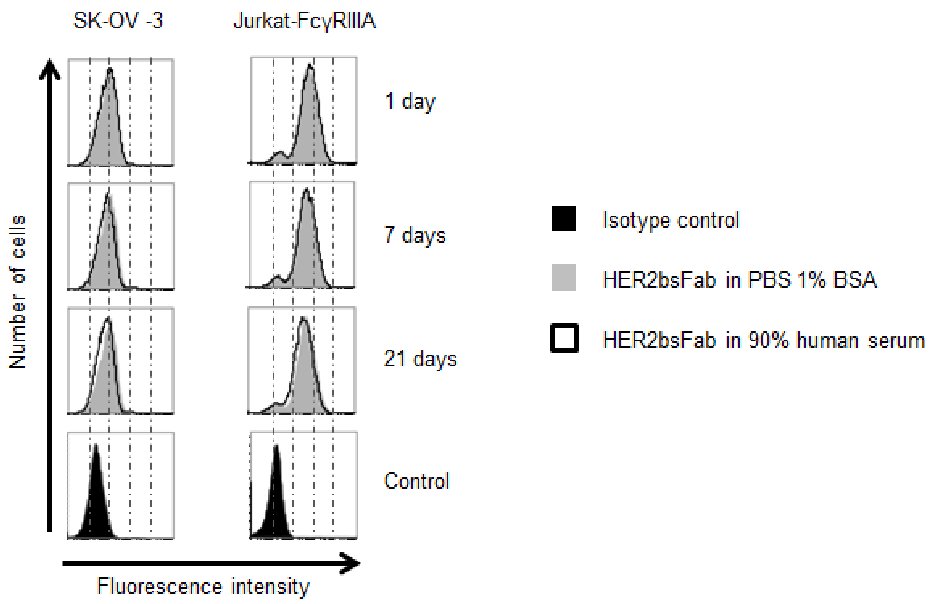

Supplementary Figure 1: HER2bsFab stability in human serum. After incubation in 90% serum for up to 21 days at 37°C, HER2bsFabs binding to SK-OV-3 and Jurkat-huFcγRIIIA cells was followed by flow cytometry using anti-His or anti-flag antibodies, respectively, followed by goat anti-mouse PE-labeled antibodies.

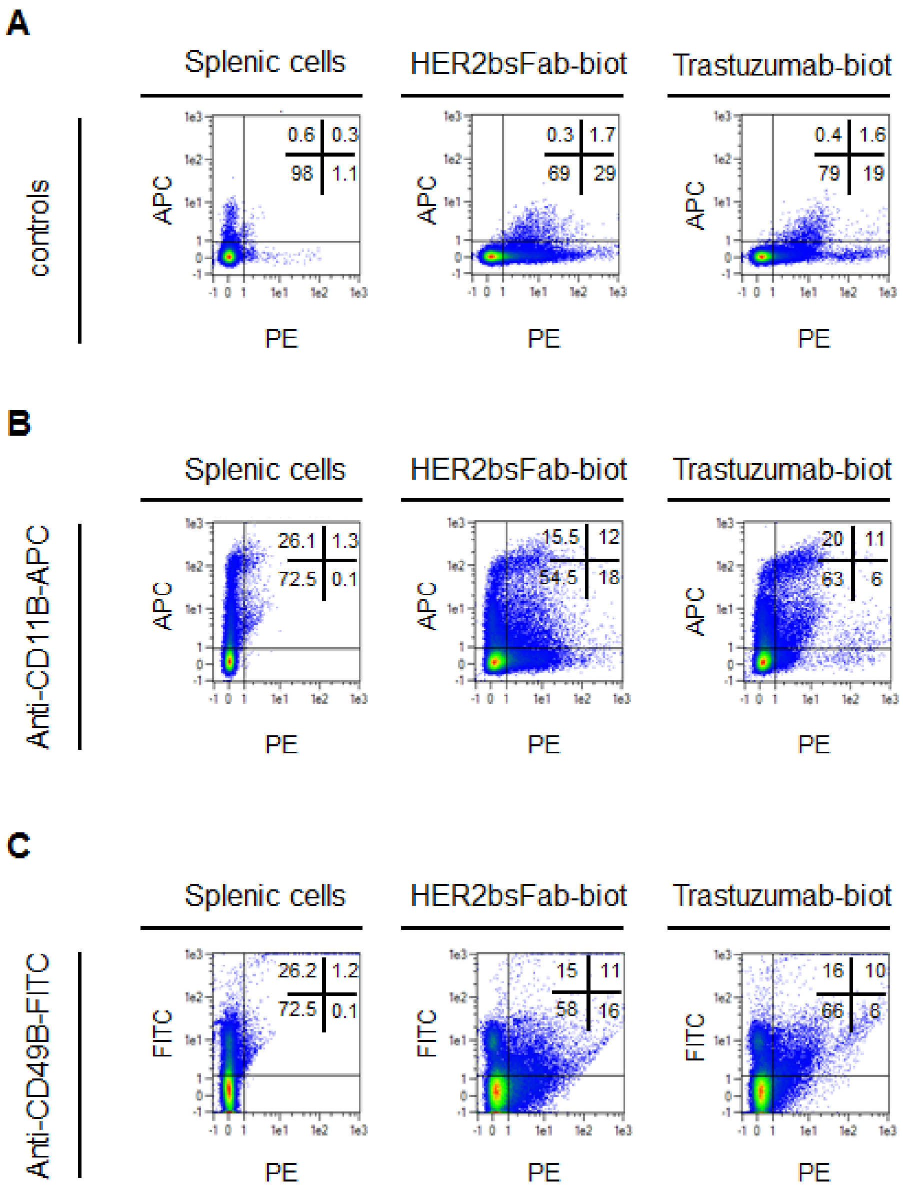

Supplementary Figure 2: Binding of HER2bsFab and trastuzumab on murine immune cells. A) Binding of biotinylated HER2bsFab (200 nM) and biotinylated trastuzumab (200 nM) was tested by flow cytometry on whole PBMC collected from two independent mouse spleens. Bound antibodies were detected using PE-labeled streptavidin. B) Binding of biotinylated HER2bsFab (200 nM) and biotinylated trastuzumab (200 nM) was tested by flow cytometry on macrophages collected from two independent mouse spleens. Bound antibodies were detected using PE-labeled streptavidin and macrophages were detected using goat anti-CD11B APC-labeled antibodies. C) Binding of biotinylated HER2bsFab (200 nM) and biotinylated trastuzumab (200 nM) was tested by flow cytometry on NK, NKT and LTγδ collected from two independent mouse spleens. Bound antibodies were detected using PE-labeled streptavidin and CD49B+ cells were detected using goat anti-CD49B FITC-labeled antibodies. Percentages of positive cells were illustrated in the upper right corner of each plot.

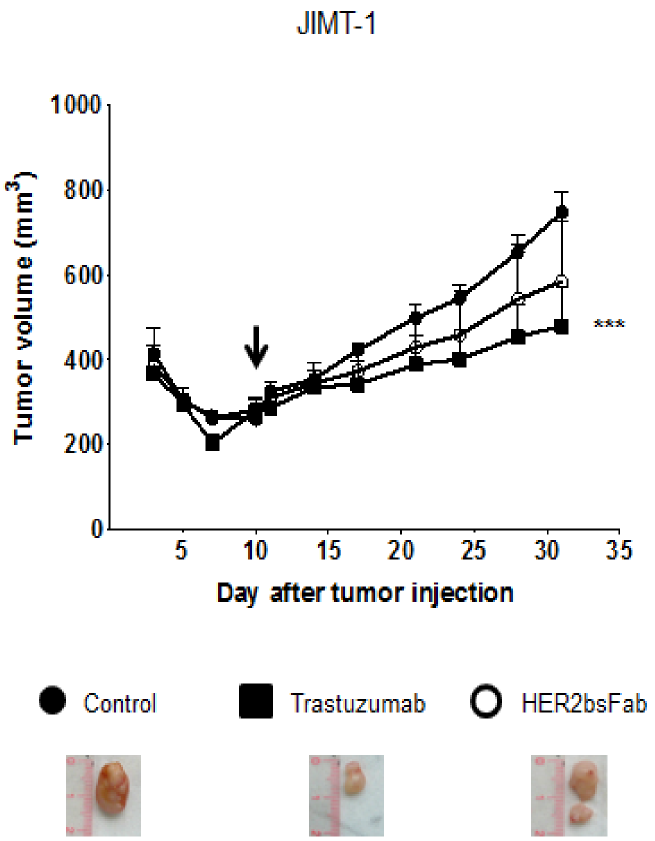

Supplementary Figure 3: *In vivo* anti-tumor activity of HER2bsFab against JIMT-1 tumors. JIMT-1 tumors were xenografted subcutaneously in NMRI Nude mice and tumor volumes were measured after treatment with HER2bsFab (5 mg/kg), PBS (control buffer) or trastuzumab (5 mg/kg). Values with error bars represent mean ± SEM. Data were analyzed by One way ANOVA test. *** $P < 0.001$ vs. control buffer group. Beginnings of treatments were illustrated by the black arrow. Representative images of tumors treated with negative controls, trastuzumab or HER2bsFab are illustrated.

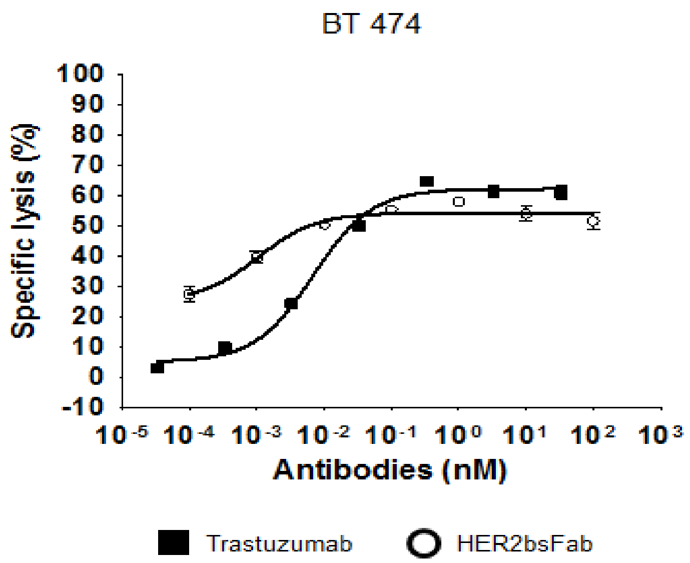

Supplementary Figure 4: HER2bsFab mediates potent *in vitro* PBMC-mediated lysis against high-HER2-overexpressing cells. ADCC assay was performed using BT 474 cells as target cells and human PBMC from 20 different donors as effector cells (E/T 25:1). Target cell viability was measured by LDH assay.

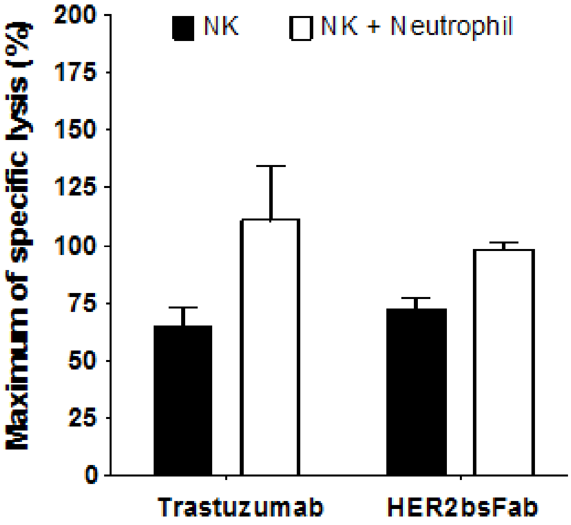

Supplementary Figure 5: Impact of neutrophils on ADCC activities mediated by HER2bsFab and trastuzumab. ADCC assays were performed using human NK cells as effector cells and BT 474 as target cells (E/T 10:1) in the presence of neutrophils (neutrophils/NK 10:1) and antibodies (10 nM). Target cell viability was measured by CellTiter-Glo viability assay. Mean values ± SEM are presented.
